# Prevalence, Infection Intensity, and Risk Factors for Soil-transmitted Helminth Infections among School Children in Northwestern Tanzania

**DOI:** 10.3390/pathogens13080627

**Published:** 2024-07-27

**Authors:** Nyanda C. Justine, Jeffer Bhuko, Sarah L. Rubagumya, Namanya S. Basinda, Deodatus M. Ruganuza, Maria M. Zinga, Matthieu Briet, Vyacheslav R. Misko, Filip Legein, Hussein Mohamed, Vivian Mushi, Donath S. Tarimo, Humphrey D. Mazigo, Wim De Malsche

**Affiliations:** 1 Department of Medical Parasitology and Entomology, Weill Bugando School of Medicine, Catholic University of Health and Allied Sciences, Mwanza P.O. Box 1464, Tanzania; jeffbhuko@bugando.ac.tz (J.B.); ruganuza@bugando.ac.tz (D.M.R.); mariazinga@bugando.ac.tz (M.M.Z.); humphreymazigo@bugando.ac.tz (H.D.M.); 2 Department of Microbiology, Immunology and Parasitology, St. Joseph College of Health and Allied Sciences, St. Joseph University in Tanzania, Dar es Salaam P.O. Box 11007, Tanzania; sara.rubagumya@sjchs.sjuit.ac.tz; 3 Department of Community Medicine, School of Public Health, Catholic University of Health and Allied Sciences, Mwanza P.O. Box 1464, Tanzania; n.basinda@bugando.ac.tz; 4 µFlow Group, Department of Bioengineering Sciences, Vrije Universiteit Brussel, 1050 Brussels, Belgium; matthieu.briet@vub.be (M.B.); veaceslav.misco@vub.be (V.R.M.); filip.legein@vub.be (F.L.); wim.de.malsche@vub.be (W.D.M.); 5 Department of Environmental and Occupational Health, School of Public Health and Social Sciences, Muhimbili University of Health and Allied Sciences, Dar es Salaam P.O. Box 65001, Tanzania; hmohameds1@gmail.com; 6 Department of Parasitology and Medical Entomology, School of Public Health and Social Sciences, Muhimbili University of Health and Allied Sciences, Dar es Salaam P.O. Box 65001, Tanzania; vmushi31@gmail.com (V.M.); dontarimo@gmail.com (D.S.T.); 7 Department of Zoology and Wildlife Conservation, College of Natural and Applied Sciences, University of Dar es Salaam, Dar es Salaam P.O. Box 35065, Tanzania

**Keywords:** prevalence, soil-transmitted helminthiasis, risk factors, school children, northwestern Tanzania

## Abstract

Soil-transmitted helminthiases (STH) are among the neglected tropical diseases and infect more than 24% of the world population. The World Health Organization recommends regular monitoring of STH’s prevalence and intensity following mass drug administrations to evaluate their effectiveness and inform future control strategies. This study evaluated the prevalence, intensity, and risk factors of STH infections among school children aged 6 to 14 years old in northwestern Tanzania. A cross-sectional study was conducted among 728 school children in the Kagera region in 2021. Participants were selected using a two-stage cluster sampling method. A questionnaire was used to collect data on the risk factors. Stool samples were examined using the Kato–Katz technique. The data were analysed using STATA. The overall prevalence of STH was 56.2% (95% CI: 52.5–59.8, 409/728). About 5.7% and 1.1% of the infected children had moderate-intensity infections with *Ascaris lumbricoides* and *Trichuris trichiura,* respectively. Risk factors included the mother’s occupation as a farmer (aOR: 1.2, *p* = 0.002) and not washing hands with water and soap (aOR: 1.4, *p* = 0.035). Washing one’s hands after using the toilet (aOR: 0.6; *p* = 0.024) is a preventive measure against STH infections. STH was prevalent in the study area. The mother’s occupation (farmer) and the lack of handwashing with water and soap influenced STH transmission. Conversely, washing hands after visiting the toilet and after playing with soil reduced the risk of STH infection.

## 1. Introduction

Globally, soil-transmitted helminthiases (STH) has been classified among the most prevalent neglected tropical diseases (NTDs) of great public health importance [1,2,3,4,5,6]. The major STH infections of global health importance are ascariasis, trichuriasis, and hookworm infections, which are caused by the nematode worms *Ascaris lumbricoides, Trichuris trichiura,* and hookworms (*Necator americanus and Ancylostoma duodenale*), respectively [4]. More than 1.5 billion people, 24% of the world’s population, were infected with STH in 2021 [7]. Infections are widely distributed in tropical and subtropical areas and affect mostly children younger than 15 years of age, contributing to 135,000 deaths annually [2]. Globally, over 270 million preschool-aged children and over 600 million school children live in areas where these parasites are intensively transmitted. These children need anthelmintic treatment, access to safe and clean water, improved sanitation, and hygiene education [3]. According to Hajare et al., the parasite *A. lumbricoides* infects 800 million people, *T. trichiura* 600 million people, and hookworm 600 million people worldwide [8]. Since these infections persist in low socioeconomic populations, they have often received less attention in the global health agenda [2,6]. The majority of the infected population lives in areas of poverty where sanitation and hygiene are inadequate [2]. The parasitic *A. lumbricoides* and *T. trichiura* infections are transmitted through the faecal–oral route by ingestion of parasitic eggs through contaminated foods, fruits, vegetables, and water. Hookworm infective larvae actively penetrate the skin when humans walk barefoot on contaminated soil [6]. In the adult stages, *A. lumbricoides* and hookworms live in the small intestine, while *T. trichiura* lives in the large intestine for years, reproducing thousands of eggs which pass through human faeces and contaminate the external environment [9]. Eggs can remain viable in the soil for several months, and larvae can remain viable for several weeks depending on prevailing environmental conditions [10]. Infections result in symptoms including diarrhoea, vomiting, anaemia, and dehydration [2]. These symptoms can subsequently lead to delayed growth and diminished cognitive function [4]. Additionally, STH infections can cause malnutrition, decreased physical fitness, and intestinal obstruction [4]. Although these infections are not usually directly fatal, they can indirectly contribute to mortality by worsening existing health conditions. Over time, these negative health effects can accumulate, leading to school absenteeism and, subsequently, poorer academic performance in children [11].

Tanzania adopted the strategies for STH control from 2004/2005 [12]. This strategy involves mass drug administration (MDA), primarily using albendazole and mebendazole, for preschool and school children who are at risk [13]. The World Health Organization (WHO) recommends annual treatment if the community’s baseline STH prevalence exceeds 20%, and biannual treatment if the prevalence exceeds 50% [12]. The WHO’s global target is to eliminate STH-related morbidities in children by 2030, which targets treatment with a coverage of 75% of children consistently in areas where STH is endemic [14]. In addition to MDA, other strategies include the provision of adequate and safe water, sanitation facilities, and hygiene education [14,15]. However, for more than a decade, the primary focus of these treatments has been school children [16,17,18]. Despite these initiatives, studies in northwestern Tanzania have reported the Kagera region as among the endemic areas for STH [19,20,21]. Little is known about the persistence of STH infection in this area, especially in the vulnerable group of school children. Therefore, this study seeks to address this gap by evaluating the prevalence, intensity, and predictors of STH infection among school children following more than a decade of MDA. Monitoring the effectiveness of the STH control strategies, as well as identifying the factors driving transmission, will equip us with the knowledge necessary to devise more effective STH control plans.

## 2. Materials and Methods

### 2.1. Ethical Approval

Ethical approval for the study was granted by the National Ethical Committee and the Lake Zone Institutional Review Board (MR/53/100/636). The study also received approval from the Muhimbili University of Health and Allied Sciences Ethical Review Board (MUHAS-REC-03-2022-1017). Further permission was obtained from the Regional and District Administrative Authorities, including those of the Muleba district and the Kagera region. Before sample collection, parents and guardians were provided with informed consent forms translated into the Kiswahili language. They were allowed to read these forms and decide whether their children would participate in the study. A thumbprint was provided for illiterate parents/guardians. Strict confidentiality was maintained for all collected information. After sample collection, all participating children were administered albendazole (400 mg) irrespective of their infection status.

### 2.2. Study Area

**Figure 1:**
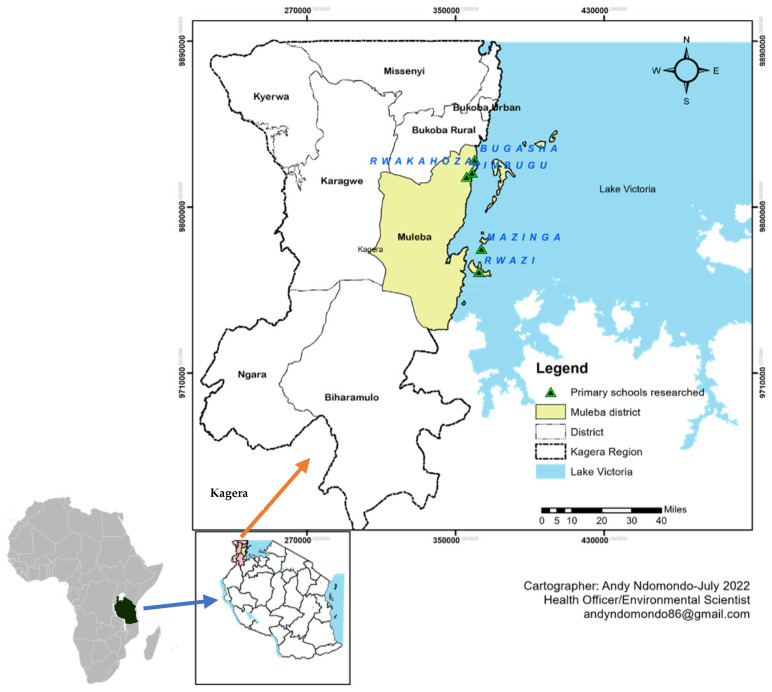
Map of Africa, Tanzania, and the Kagera region showing the location of Muleba and the study sites (primary schools). Source: (https://de.wikipedia.org/wiki/Datei:Locator_map_of_Tanzania_in_Africa.svg, accessed on 10 April 2024).

This study was conducted in the Muleba district, which is situated in the highlands of Lake Victoria within the Kagera region. Muleba, located at 1°45′ N, 31°40′ E in the northwestern part of Tanzania, in East Africa, spans an area of 10,739 km^2^, with Lake Victoria covering 62% of this area [13]. The district’s altitude ranges between 1200 m and 1500 m above sea level. The area is endemic for STH, particularly in areas at higher altitudes [13]. As per the 2022 National Census of Tanzania, the estimated population of Muleba district stands at 637,659 individuals. This includes 315,073 men and 322,586 women [22]. The primary strategy for controlling STH is the annual MDA of albendazole, which primarily targets school children. The district has 232 primary schools. The schools that participated in this study were Bugasha, Kimbugu, Mazinga, Rwakahoza, and Rwazi, all located within the Muleba district in the Kagera region (Figure 1).

### 2.3. Study Design, Population, and Inclusion and Exclusion Criteria

A cross-sectional study was conducted from April to June 2022 to determine the prevalence, intensity, and predictors of STH infection among primary school children in the Muleba district, northwestern Tanzania. Inclusion criteria were the following: primary school children aged 6–14 years from selected schools in the Muleba district, children who belonged to a school that had not received albendazole MDA in the preceding six months according to the MDA reports available at the school, and who were willing to provide stool samples on the day of data collection. The study excluded children who could not communicate due to various medical conditions and those lacking stool samples.

### 2.4. Sample Size and Sampling

The minimum sample size was calculated using the Leslie Kish formula [23]: n = Z^2^*p* (1 − *p*)/Ꜫ^2^, where n is the minimum sample size, Z is the standard normal deviation of 1.96 at the 95% confidence interval, and *p* is the expected prevalence of STH among primary school children in Kagera (39%) [21]. Ꜫ = margin of error, which was settled at 5%. We used a two-stage cluster sampling method for our study. Therefore, we factored in a design effect of 2 to account for the increased variability due to the clustering. We also anticipated a non-response rate of 26.5% to account for potential participants who either refused to participate or could not be reached. After considering all these factors, we estimated a minimum sample size of 729 participants. Sampling for participants from the Muleba district employed a two-stage cluster sampling technique. This was done as follows: In the first stage, by using the list of all 232 primary schools in Muleba, 5 schools were selected randomly as the clusters. In the second stage, at each cluster (school), a simple random sampling technique was used to select a minimum of 200 students (100 boys and 100 girls) from class one to class six from each school. The WHO recommends the selection of a total of 200–250 school children for each ecologically homogenous area to evaluate the prevalence and intensity of infection for surveys aimed at assessing the need for control measures [24].

### 2.5. Data Collection Methods

#### 2.5.1. Participant Interviews Using a Questionnaire

The research team conducted face-to-face interviews with each participant using a pretested questionnaire, which was administered in Kiswahili. The questionnaire consisted of multiple-choice, closed-ended questions. These questions were designed to collect data on various aspects including the participants’ sociodemographic information, their drinking water sources at home, sanitation factors such as toilet use, and hygiene practices.

#### 2.5.2. Interviews on School Water, Sanitation Facilities, and MDA Coverage

Onsite interviews were carried out by the research team with the school headmasters. The objective was to gather a range of information, including data on the student population, sources of water, and sanitation facilities. Information was also collected on the deworming program and the education provided on infectious diseases.

#### 2.5.3. Stool Collection and Parasitological Examination

Before sample collection, the children were given brief instructions on the collection of stool samples, and they were also allowed to ask questions. The research team was divided into three groups, with the first group receiving a stool sample and assigning unique identification numbers to the children and their sample. Children were directed to the latrine to deposit and collect a fresh sample. Stool sample containers were handed out to each child, along with a paper towel and wooden scoop for self-sample collection. The first group at the stool collection station was divided into two substations, one for girls and one for boys, where the children returned their stool samples to the research team and washed their hands. The second group managed the registration station where the children’s sociodemographic information was recorded. The third group were in charge of the sample testing. The samples were processed, and four slides for Kato–Katz analysis were prepared from the stool sample using 41.7 mg of the template and examined under a microscope within one hour for the hookworms. The slides and samples were carefully stored and packaged for transport to the Catholic University Testing Laboratory in Mwanza. At this laboratory, the Kato–Katz slides were examined under a microscope to identify the presence of *T. trichiura* and *A. lumbricoides* eggs.

#### 2.5.4. Quality Control

For quality assurance, the samples were preserved and stored to ensure their integrity. Reagents and instruments were checked for their quality before starting the lab work. The samples were inspected by their unique identification number and evaluated for quality. This quality check included assessing the volume of each sample and checking for any leakage. Ten percent of all Kato–Katz thick smears were re-examined by a third laboratory technician, who was deprived of the results of the previous two laboratory technicians. In the case of any discrepancy, the samples were reanalysed, and the results were discussed by the three technicians. The data were cleaned for any duplication of responses, errors, or invalid values.

### 2.6. Statistical Data Analysis

The data were examined to ensure completeness and to identify any missing information. Then, they were double-entered and cleaned using Microsoft Excel before being exported for analysis in STATA version 15 (StataCorp, 2017, Stata Statistical Software: Release 15. College Station, TX, USA: StataCorp LLC). The overall prevalence of STH was computed as the proportion of participants whose stool sample tested positive for at least one STH egg(s).

The intensity of STH infection was determined by calculating the Geometric Mean Intensity (GMI) of the egg counts. This was done by averaging the egg counts from four Kato–Katz smears for each individual, and then multiplying this average by 24 to obtain the number of STH eggs per gram of faeces (EPG). Based on these calculations, the intensity of the infection was then classified as light, moderate, or heavy, following the standards set by the WHO [1].

An infection with *A. lumbricoides* was classified as light if there were 1–4999 EPG, moderate if there were 5000–49999 EPG, and heavy if there were more than 50,000 EPG. Similarly, for *T. trichiura,* a light infection corresponded to 1–999 EPG, moderate to 1000–9999 EPG, and heavy to more than 10,000 EPG. For hookworms, the classifications were as follows: light intensity was defined as 1–1999 EPG, moderate intensity was defined as 2000–3999 EPG, and heavy intensity was defined as more than 4000 EPG. A *t*-test was used to compare the geometric mean of STH infections between males and females. Additionally, Spearman’s correlation was used to examine the association between the intensity of STH infections and age.

Descriptive statistics were used to summarize the sociodemographic characteristics of the participants, with frequencies and percentages reported for the categorical variables. For numerical variables such as age and family size, means and standard deviations were used for normally distributed data, while medians and interquartile ranges were used for non-normally distributed data.The chi-square test was used to test the associations between independent variables (sociodemographics of school children, personal hygiene factors, sanitation factors, and water factors) and the outcome variable (STH infection status). Finally, logistic regression was used to determine the strength of the associations between independent variables and the outcome variable. A cut-off *p*-value of 0.25 was used to determine the variables that should be considered for multivariate analysis. The model provided a crude odds ratio (cOR) for each variable added at a time and an adjusted odds ratio (aOR) after adjusting for other factors (confounders). A *p*-value less than 0.05 was considered to indicate statistical significance.

## 3. Results

### 3.1. Sociodemographic Characteristics of the Study Participants

A total of 728 primary school children with a mean age of 9.1 ± 1.9 years were enrolled. Half of these, 50.1%, were girls. The parents of most of these children were farmers, with 59.3% being fathers and 72.7% being mothers (Table 1).

### 3.2. Prevalence and Intensity of STH among Primary School Children

#### 3.2.1. Prevalence of STH among Primary School Children

The overall prevalence of STH among primary school children was 56.2% (409/728, 95% CI: 52.5–59.8). The results of the KK smear test indicated that 40.8% (297/728, 95% CI: 37.2–44.4) of the children were infected with *A. lumbricoides*, 36.7% (267/728, 95% CI: 33.2–40.3) were infected with *T. trichiura*, and there were no cases of hookworm infection (0.0%) among the children. *A. lumbricoides* and *T. trichiura* coinfections were observed in 21.3% (155/728, 95% CI: 18.4–24.4) of the children.

The overall prevalence of STH in girls was 57.3% (209/365, 95% CI: 52–62.3). Among all the schools visited, Mazinga had an overall STH prevalence of 69.9% (107/153, 95% CI: 62–77.1) (Table 2).

#### 3.2.2. Intensity of STH Infection among Primary School Children

The overall geometrical mean egg count for *A. lumbricoides* among infected children was 323.3 EPG (95% CI: 258.9–403.8), and that for *T. Trichiura* was 41.8 EPG (95% CI: 36.3–48.1). The geometrical mean egg count for *A. lumbricoides* among girls was 380.1 EPG (95% CI: 278.2–519.3) and it was 269.7 EPG (95% CI: 196.3–370.6) among boys. *T. trichiura*-infected girls had a mean geometrical faecal egg count of 45.3 EPG (95% CI: 38.3–54.9), and it was 38.5 EPG (95% CI: 31.3–47.2) for boys. Age showed a non-significant negative correlation with *A. lumbricoides* infection intensity (rs = −0.0260, *p* = 0.4841), and a non-significant positive correlation with *T. trichiura* infection intensity (rs = 0.0617, *p* = 0.0961) among primary school children. See Table 3.

#### 3.2.3. STH Infection Intensity Categories

The majority of the infected participants had light-intensity infections, 94.3% (95% CI: 90.6–96.6, 280/297) with *A. lumbricoides* and 98.9% (95% CI: 96.8–99.8, 264/267) with *T. trichiura*. Of all the participants infected with *A. lumbricoides,* 5.7% (95% CI: 3.4–9.0, 17/297) were classified as moderate-intensity infections. Similarly, in the case of *T. trichiura*, 1.1% (3/267, 95% CI: 0.2–3.2) were found to be of moderate intensity. In all infected children, no heavy-intensity infections were observed. Thus, of all the study participants, 2.7% (95% CI: 1.7–4.2, 20/728) had a moderate intensity of STH infection.

### 3.3. Factors Associated with STH among Primary School Children

According to the multivariate analysis, the children’s mothers’ occupations, washing hands with water and soap, and washing hands after visiting the toilet were the independent factors associated with STH infection. The children whose mothers were farmers had 1.2 times higher odds of having an STH infection compared to children whose mothers were involved in fishing (aOR: 1.2, 95% CI: 0.9–1.7). The children who reported not washing their hands with water and soap had 1.4 times higher odds of STH infection than the children who reported washing their hands with water and soap (aOR: 1.4, 95% CI: 1.0–2.1). Compared with those who did not wash their hands, children who washed their hands after visiting the toilet had 0.6 times lower odds of STH infection (aOR: 0.6, 95% CI: 0.4–0.9) (Table 4).

### 3.4. School Water Source, Sanitation Facilities, and MDA Coverage

A significant majority (four out of five) of the schools relied on river water for their daily activities. Each of the visited schools had a latrine located within the school premises. The types of latrines varied: one school utilized a simple pit latrine, three schools had improved latrines, one of which was a ventilated pit latrine, and two schools were equipped with flush toilets. All five schools confirmed that they educate their students about infectious diseases. However, only two out of five schools (40%) included information about intestinal worms in their curricula. Three of the five schools introduced this topic to students from the third grade onwards. All the schools participated in the MDA programs, including the most recent round, which was conducted six months before our study. The uptake of the last MDA round exceeded the recommended coverage in all schools: 97% for Bugasha, 100% for Kimbugu, 85% for Mazinga, 98% for Rwakahoza, and 97% for Rwazi. This indicates a high level of participation in these important health programs.

## 4. Discussion

### 4.1. Prevalence and Intensity of STH

The current study provides insight into the distribution of STH in northwestern Tanzania. The study revealed that the overall prevalence of STH was 56.2%, with 5.7% and 1.1% of the children having moderate-intensity infections with *A. lumbricoides* and *T. trichiura*, respectively. The study also revealed that the mother’s occupation as a farmer and not washing their hands with water and soap were risk factors associated with STH infection among primary school children. Washing hands after visiting the toilet and washing hands after playing with soil were found to be protective against STH infection.

The findings from this study add to the growing evidence that school children are the most highly affected population [25]. The overall prevalence of 56.2% in the most treated group was unacceptably high, especially in the era of preventive chemotherapy, and when the WHO targets are moving to the elimination phase [14]. However, this prevalence seems to decrease with age, similar to the findings from Ethiopia [26]. The gender difference was not significant, but girls seemed to be more affected than boys. The overall prevalence of STH observed in this study was comparable to the 57.2% reported among school children in the same age group in Indonesia [27]. In contrast to our findings, other studies have reported a lower prevalence of STH in the Sengerema, Ukara Island, and Ngorongoro conservation areas, with prevalences of 6.7%, 10.88%, and 32.3%, respectively [5,28,29]. The observed differences could be due to several factors, including but not limited to the level of hygiene, water used for drinking, and type of toilet facility, which vary among these study areas. However, environmental factors such as humid weather and wet soil due to rainfall, which are favourable for the STH life cycle, could explain the abundance of STH in the study area [19].

The WHO has set a goal to eliminate STH infections as a public health problem by the year 2030. This is defined in their revised Neglected Tropical Disease roadmap for 2021–2030 as having less than 2% moderate- and heavy-intensity STH infections [14]. A large-scale MDA intervention is recommended when the infection rate in school-aged children (SAC) reaches or exceeds 20% [14]. In communities where the prevalence is 50% or higher, the WHO advises that all school children should be treated twice a year, regardless of whether they are enrolled in school [14]. If resources permit, treatment can be increased to three times a year [12,14]. The recommendation is to treat school children once a year in communities where the prevalence is between 20% and 50% [12,14]. This approach is designed to control and eventually eliminate STH infections effectively [30]. These findings show that the study area is classified as a high-risk area (prevalence ≥ 50%), in which the WHO recommends treatment of school children twice a year [31].

In this study, we found that 21.3% of the children were coinfected with *A. lumbricoides* and *T. trichiura*. This prevalence of STH coinfection was higher than the 0.9% reported in Bagamoyo, Tanzania [32]. The difference could be explained by the geographical and environmental conditions. Muleba, unlike Bagamoyo, is characterized by wet soil and humid conditions, which persist for extended periods throughout the year [20]. These conditions create a conducive environment for the transmission of STH, thereby explaining the higher prevalence of coinfection in this region. However, our findings are consistent with a report from Laguna Province in the Philippines, where 25% of coinfections were observed [33]. It is important to note that STH coinfections are common and can pose a significant health risk to children, even at lower prevalence rates [34]. These coinfections have implications for current STH treatment guidelines, as individuals with multiple STH infections tend to experience more severe morbidities. A common feature of STH infections is that these parasites share similarities in their life cycle and geographical distribution, which allows them to be controlled by similar approaches. The health impacts of STH coinfections are wide-ranging and include malnutrition, anaemia, stunting, and poor cognitive development [35]. Furthermore, school absenteeism, a common issue associated with STH infection, has been reported among school children in sub-Saharan Africa (SSA). This highlights the need for comprehensive and effective treatment and control strategies to address the challenge of STH infections [36].

In this study, *A. lumbricoides* was the most dominant STH species at 40.8%, followed by *T. trichiura* at 36.7%. These results are similar to previous findings in Indonesia (40.1%) [25] and Kerewo Town, Ethiopia (41.2%) [26], but are different from those of studies in Ukara Tanzania [28] and Sengerema, Tanzania [20], where hookworms were identified as the prevalent species. The prevalence of hookworms in Ukara and Sengerema could be attributed to the sandy soil, which is favourable for these parasites. In contrast, Muleba has a mix of sandy, clay, and loamy soil [28]. Moreover, the decreasing prevalence of hookworms observed over the past decade could reflect the impact of ongoing MDA and other control programs in northwestern Tanzania [21]. However, the 0% prevalence of hookworm infection observed in this study does not unequivocally imply the absence of such infections within the study area. Our results are contrary to the findings reported in a precision mapping study in northwestern Tanzania, where a prevalence rate of 6.2% for hookworm infection was identified among school children [19]. This discrepancy could potentially be attributed to the difference in sample sizes. Our study incorporated a sample size of 728 participants, in contrast to the 8698 participants involved in the precision mapping study [19]. Moreover, the diagnostic method used may have contributed to this finding, as the Kato–Katz test has limited sensitivity in detecting hookworms, especially in light-intensity infections [37,38]. Although studies have suggested testing for novel diagnostics for STH to counter this limitation, Kato–Katz remains the WHO gold standard for evaluating STH infections [7,13,39].

The majority of infections ranged from light to moderate in intensity, with no reported cases of heavy infections. This trend is consistent with findings from other studies in Tanzania, including a study from Ukara Island [28], as well as studies from Rwanda [40] and Indonesia [27]. These results underscore the positive impact of ongoing MDA programs. Furthermore, they indicate that Tanzania is making significant progress towards the WHO’s objective of eliminating STH as a public health problem.

### 4.2. Factors Associated with STH Infection among School Children

One of the factors influencing STH infection was the mother’s occupation. Having a mother who is a farmer increased the risk of STH infection among school children. These findings corroborate the findings from a study conducted in Nigeria [41], although contrary to this study, studies performed in Ethiopia [42] reported no association between parents’ occupations and STH infection in their children. In the study area, agriculture is the main occupation. However, the lack of toilets on farms, where children frequently help their mothers, increases their exposure to contaminated soil and promotes open defecation among both adults and children. This risk is heightened by the absence of sanitation and handwashing facilities in these farming environments, where families spend a significant amount of time. This scenario presents a significant challenge to control programs, particularly in implementing water, sanitation, and hygiene practices for STH control. Our study found that washing hands with soap and water was a protective measure against STH infection, a finding which aligns with a similar study conducted in Bagamoyo, Tanzania [32]. This correlation is further supported by the findings from Ethiopia [26] and Indonesia [27]. In addition to this, we discovered that the practice of washing hands after using the toilet significantly reduced the risk of STH infection. Interestingly, this observation is consistent with a study carried out in Kenya [43], further emphasizing the importance of hygiene practices in controlling STH infection. Handwashing with soap and water after defecation effectively removes residual faecal material from one’s hands, thereby reducing the risk of ingesting it with food and, consequently, the risk of STH transmission. This aligns with studies conducted in North Sumatra, Indonesia [27], and western Ethiopia [26]. This simple yet crucial practice can serve as a significant preventive measure against STH infection.

In this study, 5.4% of participants practised open defecation. Although this percentage is small, it still signifies a potential risk for soil contamination and STH transmission in the area. However, no significant association was found between this practice and STH infection (*p* > 0.05), corroborating the findings from Nigeria [41]. The prevalence of STH was higher among pit latrine users than among those using pour/flush toilets. This finding is consistent with a study from Nigeria, which revealed a greater risk of STH infection among students who used pit latrines at home [41]. If not properly maintained, pit latrines can become breeding grounds for houseflies, which can then transport helminth eggs to food and fruits, thereby increasing the risk of STH transmission [44].

This study revealed that intestinal worms were included in the curricula of only two out of five schools. This suggests that most participants lacked knowledge about STH. Despite the importance of health education in controlling STH infection, a clinical trial in Kagera showed that an intervention that combined education and resources to encourage handwashing in schools and homes failed to significantly reduce STH infections [21]. The issue could be due to the influence of regular classroom teaching on children’s behaviour. A similar challenge of integrating classroom-taught knowledge on water, sanitation, and hygiene into practice was identified among school children in Ngorongoro, Tanzania [5]. We call for more studies to explore this relationship further.

The annual MDA practice has proven effective in reducing the prevalence of heavy-intensity infections, as demonstrated by this study. However, the overall prevalence remains high, potentially due to various factors driving the transmission. We hypothesize that the observed prevalence could be attributed to children who miss MDA campaigns, those not covered by MDA and re-infections. Additionally, as treatments are primarily targeted at children, adults may act as a reservoir for infection, particularly in areas without ongoing lymphatic filariasis control programs. This hypothesis is supported by a recent study in Muleba, which found evidence of soil-transmitted helminth (STH) transmission among adults [20]. These findings underscore the need to expand treatment to other demographic groups and increase coverage among school children to effectively control the spread of STH.

## 5. Study Limitations

This study, while insightful, faced certain limitations. We collected a single stool sample per child per day, rather than the recommended practice of three consecutive daily samples. This could lead to an underestimation of disease prevalence. To mitigate this, we prepared four Kato–Katz smears per sample and employed skilled laboratory technicians for examination. The limited sensitivity of the Kato–Katz technique, particularly for hookworm detection, may have resulted in the observed 0% hookworm prevalence. Although the cross-sectional nature of our study precludes establishing causality, it allows us to identify associations and provide a snapshot of the magnitude, predictors, and implications of STH infection. This information is vital for the planning and execution of effective STH control programs.

## 6. Conclusions

Despite a decade of preventive chemotherapy through the school MDA program, our study revealed a high prevalence of STH infections among school children. Key predictors of STH infection included the parents’ occupations and hygiene practices, such as handwashing with soap and water, particularly after using the toilet. As a result, we recommend that the control program administer MDA twice yearly to school children and emphasize hygiene education, focusing on frequent handwashing and proper sanitation. The program should also integrate MDA with the provision of handwashing facilities and improved sanitation in schools. To ensure sustainable STH control, further community-level studies are recommended to assess the prevalence thresholds and treatment needs.

## Figures and Tables

**Table 1:** Sociodemographic characteristics of the study participants (n = 728).

Variable	Category	Frequency (Percent)
Age (Years)	(Mean ± SD) 9.1 ± 1.9	
Gender	Males	363 (49.9)
	Females	365 (50.1)
Family Size	Median (IQR): 6 (5–8)	
Schools	Kimbugu	133 (18.3)
	Mazinga	153 (21.0)
	Rwakahoza	135 (18.5)
	Rwazi	140 (19.2)
	Bugasha	167 (22.9)
Mother’s Occupation	Farmer	529 (72.7)
	Livestock keeper	2 (0.3)
	Fishing	17 (2.3)
	Employed	20 (2.7)
	Small businesswoman	145 (19.9)
	Others	15 (2.1)
Father’s Occupation	Farmer	432 (59.3)
	Livestock keeper	35 (4.8)
	Fishing	185 (25.4)
	Employed	31 (4.3)
	Small businessman	21 (2.9)
	Others	24 (3.3)

**Table 2:** The overall prevalence of STH among primary school children by gender and school (n = 728).

Variable	Category	Total (%)	Prevalence n (%)
Gender	Males	363 (49.9)	200 (55.1)
	Females	365 (50.1)	209 (57.3)
Schools	Kimbugu	133 (18.3)	72 (54.1)
	Mazinga	153 (21)	107 (69.9)
	Rwakahoza	135 (18.5)	63 (46.7)
	Rwazi	140 (19.2)	36 (25.7)
	Bugasha	167 (22.9)	131 (78.4)

**Table 3:** Intensity of STH infection among primary school children by age and sex.

STH	Intensity	Geometric Mean (EPG) (95% CI)	Statistical Test
*A. lumbricoides*	Overall	323.3 (258.9–403.8)	
	Age		rs = −0.0260, *p*= 0.4841
	Male	269.7 (196.3–370.6)	
	Female	380.1 (278.2–519.3)	t = 2.4902, *p*= 0.0130
*T. trichiura*	Overall	41.8 (36.3–48.1)	
	Age		rs =0.0617, *p* = 0.0961
	Male	38.5 (31.3–47.2)	
	Female	45.3 (37.3–54.9)	t = 0.8587, *p*= 0.3908

**Table 4:** Multivariate analysis of factors associated with STH infection.

Factor	n (%)	STH Positive n (%)	cOR (95% CI)	*p*-Value	aOR (95% CI)	*p*-Value
Age of children (years)			1.0 (0.9–1.11)	0.479	1.1 (0.9–1.2)	0.216
Mother’s occupation						
Farmer	529 (72.7)	369 (69.7)	2.1 (1.4–3.2)	0.001	1.2 (0.9–1.7)	0.002 *
Small businesswoman	145 (19.9)	71 (48.9)	0.7 (0.5–1.1)	0.099	0.8 (0.5–1.2)	0.280
Others	15 (2.1)	3 (0.20)	1.2 (0.2–7.1)	0.863	1.9 (0.3–12.4)	0.463
Employed	20 (2.7)	2 (0.1)	0.8 (0.3–6.2)	0.734	0.9 (0.4–5.7)	0.137
Fishing	17 (2.3)	13 (76.5)	1		1	
Washing hands with water and soap						
No	541 (75.4)	316 (58.4)	1.2 (1.0–1.4)	0.048	1.4 (1.0–2.1)	0.035 *
Yes	176 (24.5)	87 (49.4)	1		1	
Washing hands after visiting the toilet						
No	12 (1.6)	4 (33.3)	1		1	
Yes	714 (98)	258 (36.1)	0.8 (0.6–1.1)	0.123	0.6 (0.4–0.9)	0.024 *
Washing hands after playing with soil						
No	135 (20.6)	83 (61.5)	1		1	
Yes	521 (79.4)	288 (55.3)	0.8 (0.7–1.1)	0.177	0.7 (0.5–1.1)	0.144
Type of toilet at home						
Pour/flush toilet	68 (9.5)	32 (47.1)	1		1	
Pit latrine	645 (90.5)	370 (57.4)	1.1 (0.8–1.5)	0.137	1.5 (0.9–2.6)	0.082
Regularly use the toilet at home						
No	63 (8.7)	29 (46.0)	1		1	
Yes	665 (91.3)	380 (57.1)	1.2 (0.9–1.6)	0.124	1.3 (0.8–2.3)	0.328

cOR stands for crude odds ratio, aOR stands for adjusted odds ratio; * statistically significant at *p* < 0.05.

## Data Availability

All relevant data for the study are available upon reasonable request.
